# Supplementary figures and images for: Increased oxidative stress in elderly leprosy patients is related to age but not to bacillary load
Source: PLoS Negl Trop Dis. 2021 Mar 9;15(3):e0009214. doi: 10.1371/journal.pntd.0009214 (PMC7978340; doi:10.1371/journal.pntd.0009214)

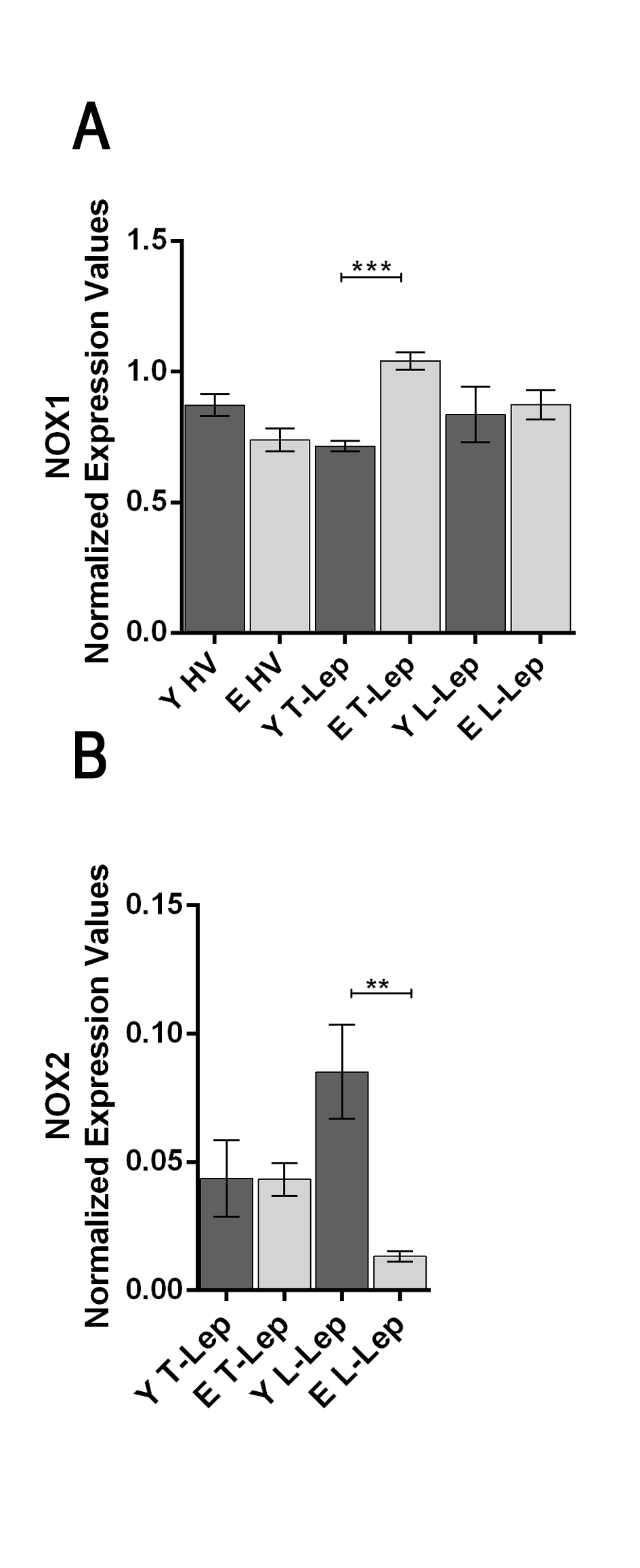

Supplement: S1 Fig — Quantitative PCR (qPCR) evaluation of NOX1 mRNA levels (A) in whole blood (L-Lep patients, Y n = 12 and E n = 13; T-Lep patients Y n = 12 and E n = 15; HV Y n = 10 and E n = 15), and NOX2 (B) in skin lesion samples (Y L-Lep n = 13 and E L-Lep n = 10; Y T-Lep n = 10 and E T-Lep n = 12). Bar graphs represent means ± SD of each group. Data analysis was performed using Kruskal-Wallis test followed by Dunn’s multiple comparison post-test. **P < 0.01, and ***P < 0.001. Abbreviations: E–Elderly; Y–Young; T-Lep–TT/BT patients; L-Lep–LL/BL patients; HV–healthy volunteers. (TIF) [file pntd.0009214.s001.tif]
