# Supplementary material for: Increased oxidative stress in elderly leprosy patients is related to age but not to bacillary load
Source: PLoS Negl Trop Dis. 2021 Mar 9;15(3):e0009214. doi: 10.1371/journal.pntd.0009214 (PMC7978340; doi:10.1371/journal.pntd.0009214)
Supplement: S1 Table — (DOCX) [file pntd.0009214.s002.docx]

| ***Gene*** | **Gene name** | **Sequence 5'** 🡪 **3'** | **Gene bank** |
| --- | --- | --- | --- |
| *SOD1* | Superoxide dismutase 1 | Fwd - GGGAAGCATTAAAGGACTGACTG | NM_000454.5 |
|  |  | Rev - CCAACATGCCTCTCTTCATCC |  |
| *SOD2* | Superoxide dismutase 2 | Fwd - CAGCGGTAGCACCAGCACTA | NM_000636.4 |
|  |  | Rev - GAGCCCAGATACCCCAAAAC |  |
| *GSR* | Glutathione-disulfide reductase | Fwd - CAGCCCTGGGTTCTAAGACAT | NM_000637.5 |
|  |  | Rev - CCTTGACCTGGGAGAACTTCAG |  |
| *GPX1* | Glutathione peroxidase 1 | Fwd - GGAGAACGCCAAGAACGAAG | NM_000581.4 |
|  |  | Rev - CGCACTTCTCGAAGAGCATGA |  |
| *NOX1* | NADPH oxidase 1 | Fwd - GTTTCTGGTTGTTTGGTTAGGGC | NM_007052.5 |
|  |  | Rev - ATTGCTTTCTCAGTGTGCGG |  |
| *NOX2* | NADPH oxidase 2 | Fwd - TCCTATGACTTGGAAATGGATAGTGG | NM_000397.4 |
|  |  | Rev - CCTTCTTCTTCATCTGTAGCTCG |  |
| *RPL13* | Ribosomal protein L13 | Fwd - GACAAGAAAAAGCGGATGGT | NM_000977.4 |
|  |  | Rev - GTACTTCCAGCCAACCTCGT |  |
| *RPS16* | Ribosomal protein S16 | Fwd - GCGCACGCTACAGTACAAG | NM_001020.6 |
|  |  | Rev - AGATGGACTGACGGATAGCATA |  |
| *RPL35* | Ribosomal protein L35 | Fwd - CGAGTCGTCCGGAAATCCAT | NM_007209.4 |
|  |  | Rev - GGCTTGTACTTCTTGCCCTTG |  |

**S1 Table Oligonucleotides used in the study.**
