# Supplementary material for: Increased oxidative stress in elderly leprosy patients is related to age but not to bacillary load
Source: PLoS Negl Trop Dis. 2021 Mar 9;15(3):e0009214. doi: 10.1371/journal.pntd.0009214 (PMC7978340; doi:10.1371/journal.pntd.0009214)
Supplement: S2 Table — (DOCX) [file pntd.0009214.s003.docx]

**S2 Table Biochemical data of analyzed group.**

|  | E T-Lep | Y T-Lep | E L-Lep | Y L-Lep | E HV |
| --- | --- | --- | --- | --- | --- |
| Glucose (mg/dL) | 98.4 ± 15.4 | 97.5 ± 23 | 109 ± 39.5 | 92.6 ± 7.6 | 119.4 ± 24.5 |
| Creatinine (mg/dL) | 1.1 ± 0.3 | 0.8 ± 0.3 | 0.9 ± 0.2 | 0.9 ± 0.2 | 0.9 ± 0.2 |
| Urea (mg/dL) | 35.7 ± 10.5 | 30.3 ± 8.3 | 36.1 ± 9.2 | 26 ± 8.6 | 42.1 ± 13.6 |
| Total bilirubin (mg/dL) | 0.8 ± 0.3^b^ | 0.3 ± 0.1^b, c^ | 0.5 ± 0.1 | 0.6 ± 0.5 | 0.9 ± 0.3^c^ |
| Albumin (g/dL) | 3.7 ± 0.4 | 3 ± 0.8 | 3.8 ± 0.5 | 3.7 ± 0.7 | 3.6 ± 1.9 |
| Uric acid (mg/dL) | 5.4 ± 0.9 | 3.6 ± 2.2 | 3 ± 1.2 | 2.9 ± 1.6 | 4.3 ± 1.4 |
| Total cholesterol (mg/dL) | 176.9 ± 40.4 | 150.5 ± 42.7^a^ | 191.6 ± 24.9^a^ | 182.4 ± 36.7 | 192.9 ± 46.7 |
| Triglycerides (mg/dL) | 140.4 ± 82.8 | 112.1 ± 66.3 | 126.5 ± 56.6 | 112.1 ± 66.4 | 146.9 ± 67.1 |
| HDL (mg/dL) | 34.2 ± 8.5^a^ | 33 ± 6.1^#, b^ | 55.8 ± 15.6^a, #^ | 50.8 ± 12 | 51.8 ± 8.9^b^ |
| LDL (mg/dL) | 101.9 ± 22.7 | 82.7 ± 21.6 | 99.9 ± 36.9 | 119.8 ± 31.6 | 111.6 ± 39.2 |
| VLDL (mg/dL) | 30.2 ± 17.9 | 21.8 ± 16.8 | 25.3 ± 12.2 | 24.9 ± 11.9 | 29.5 ± 13.5 |

^a^*P* < 0.05; ^b^*P* < 0.01; ^c^*P* < 0.001; ^#^*P* <0.05
