# Supplementary material for: Increased oxidative stress in elderly leprosy patients is related to age but not to bacillary load
Source: PLoS Negl Trop Dis. 2021 Mar 9;15(3):e0009214. doi: 10.1371/journal.pntd.0009214 (PMC7978340; doi:10.1371/journal.pntd.0009214)
Supplement: Attachment — Submitted filename: Answer_to_the_referees.docx [file pntd.0009214.s004.docx]

Cover letter to the Editor

Dear Sir,

We are grateful for the opportunity to submit our revised manuscript. We thank the reviewers for careful and detail revision of our paper ID # PNTD-D-20-01951 entitled “Increased oxidative stress in elderly leprosy patients is related to age but not to bacillary load”. We have answered reviewer´s comments point-by-point below and incorporated most of the suggestions in the revised version of the text. We uploaded two versions of the manuscript: one with highlighted changes (concerning the reviewers´ issues that addresses all emends) and a clean version. We hope that the manuscript is now acceptable in the present form in this prestigious journal.

Best Regards

Danuza Esquenazi

*Corresponding author*

Reviewer #1: A few suggestions to improve the organization of the manuscript are described in the summary/general comments.

Reviewer #2: Overall, I have minor comments that should be addressed to increase rigor and clarity. A few typos need to be corrected (e.g. line 125, 126).

**We thank the reviewer for kindly drawing our attention to this topic. The corrections were performed accordingly. We withdraw “the” in the line 127, and we correct the word “impair” in the line 128.**

The order in table and figure calling is often not correct (e.g. Table S2 appears before Table S1, Fig 1E before Fig 1B...), which disturbs the flow of the article.

**We appreciate the collaboration from the reviewer. We changed the order of the Tables S1 and S2, as well as the order of appearance of Figure 1 inside the manuscript.**

-    Introduction:

o   Please give more details about the borderline forms of leprosy

**According to the reviewer’s comment, we altered the text of the manuscript by inserting data concerning the borderline forms (lines 90-92 and new reference # 4).**

o   Line 123: is it possible to provide a more recent review or article?

**We thank the reviewer for drawing our attention to the need of updating the references, and believe that the manuscripts Pomato et al., 2018 and Chocron et al., 2019 (references #25 and 26; line 125) shall be suitable for such.**

o Line 128: a hypothesis should not be conditional (whether..)

**We thank the reviewer and inform that we replaced the phrasal structure to convert the sentence into a direct statement (line 130).**

-  Methods:
o    Hypertensive and diabetic individuals were not excluded. Is there any data on ROS production in such individuals?

**The reviewer is right: the hypertensive and diabetic individuals were not excluded. Nevertheless, in our study, they did not present alterations in the oxidative damage, when compared to non-hypertensive, or to diabetic patients.**

o     Line 257: Graph Prism should be GraphPad Prism if I'm not mistaken.

**The reviewer is right, and we thank him/her. The name of the software was checked and duly corrected in the manuscript (line 257).**

-   Results:

o    Overall, barplots should be replaced by graphs displaying the distribution of the samples (by box plots or else). Also, these graphs should be presented with the same categories and the same ordering for clarity (e.g. following what's displayed on Fig 1A). In addition, I think most graphs may display the mean and not the median as it is stated. The use of distinct colors in graphs differentiating L-Lep and T-Lep individuals (and controls) would be of great help for clarity and interpretation.

**We thank the reviewer for his remarks. All the graphs from figure 1 were altered, with each individual being shown in circles or squares, and with conversion of all the values from horizontal bars into average/means. The alterations were performed in the figures. Colors were used to differentiate the various leprosy clinical forms. The same was done in figure 4.**

o Characteristics of the subjects:

The authors compared T-Lep vs L-Lep but have data separating T-Lep (TT and BT) and L-Lep (BL and LL). Why the authors do not take advantage of this classification (which could lead to more accurate conclusions)?

Table 1: What is the age stratification among TT, BT, BL and LL samples?

**Please see below the age stratification. Our sampling favored the analysis for young and elder patients. We did not observe significant difference in relation to the age range according to the leprosy clinical form. Important to mention that according to the protocol and ethics we excluded patients younger than 18y/o. So, we kept the form of the table as originally submitted, namely: the mean ± SD of ages from each group. However, since patient were not randomly selected, as a strategy to increase the number of elderly patients and trying to provide a more polarized profile we fixed recruitment of young from 20 to 40 years and elders (>60 years old). This polarization strategy was defined in way that there are some age ranges that we did not have patients. So, we have rewritten the sentence in the methods section “recruited according to age” (line 152) to better explain this issue and, we added a sentence in the discussion section addressing the limitations of the study (lines 491-497).**

o Increased oxidative damage in serum samples of elderly leprosy patients.

The paragraph starts abruptly with a conclusion, not stating the hypothesis, and how it was tackled.

**The reviewer is right. We altered the title of the section and inserted an introductory sentence for the sake of clarity (lines 286-288).**

Line 288: I observe the exact contrary to the statement made by the authors (Fig 1A).

**We appreciate the remark and apologize for our mistake. The manuscript was corrected accordingly (line 291).**

Line 305: Only E T-Lep are different from the other groups: 24-76% vs 65-35%. Could the authors give an explanation to this?

**We appreciate the remark from the reviewer. According to epidemiological studies, elderly men are at higher risk of developing multibacillary leprosy. This finding observed in the studied population is mentioned in the manuscript, being corroborated by the references #20 and 21 (lines 590-598).**

**Remark: in view of the Editor’s comment, we decided to remove the sentence “The analysis of oxidative damage between men and women does not seem to influence oxidative stress, at least under the conditions analyzed in this study (data not shown)”, from line 308 of the manuscript with HIGHLIGHTS.**

Line 310: Please, in addition to a *P* value, provide beta (or OR) and CI.

**We thank the reviewer for his remark and confirm that the requested information was inserted into the text, as follows: “BI was not statistically significant (beta=-0.762 95% IC = [-2.405;0.881], P = 0.346), while age was an independent factor associated with protein carbonyl level (beta=6.867 95% IC= [2.500; 11.234], *P* = 0.004; Lines 310-314)” These findings referred to the parameters as estimated according to a general linear model (GLM) per BI and age.**

| **Parameter Estimates of GLM analysis for protein carbonyl levels among L-Lep patients.** | | | | | | |
| --- | --- | --- | --- | --- | --- | --- |
| ***Parameter*** | ***B*** | ***Std. Error*** | ***T*** | ***Sig.*** | ***95% Confidence Interval*** | |
|  |  |  |  |  | ***Lower Bound*** | ***Upper Bound*** |
| **Intercept** | **31,365** | **3,615** | **8,676** | **,000** | **23,847** | **38,883** |
| **BI** | **-,762** | **,790** | **-,964** | **,346** | **-2,405** | **,881** |
| **[age]^a^** | **6,867** | **2,100** | **3,270** | **,004** | **2,500** | **11,234** |
|  |  |  |  |  |  |  |
| a. **reference category is the young group** | | | | |  |  |

**Remark: as requested, the expression “data not shown” was removed from the manuscript with HIGHLIGTS. Anyway, to avoid further doubts, the Table demonstrating the GLM analysis is found above.**

o Protein carbonyl concentration increases one year after MDT discharge in young L-Lep patients. Why focusing only on L-Lep patients? I would like to see the parallel with T-Lep patients (and healthy controls?).

**We thank for the remark. Nevertheless, in this topic, the purpose was to correlate BI with the oxidative damage during the studied period, and, as T-Lep patients presented BI corresponding to zero, any analysis became unfeasible. So, this section of the study was only performed with L-Lep patients.**

Line 326: changes after MDT do not seem "subtle" for each sample.

**The reviewer is right. We apologize for our mistake. The sentence was duly corrected in the manuscript (line 330).**

o    Elderly leprosy patients present higher presence of 4-HNE in skin lesion samples:

What is the conclusion of the paragraph? What to conclude about Figure 3?

**We thank the reviewer for drawing our attention to this topic. The request was complied with, and a conclusion about the experiments shown in the Figure 3 was inserted into the manuscript (lines 352-354).**

o   Lower levels of antioxidant enzymes in elderly L-Lep skin lesion:
Fig4 A and B are not commented. What about SOD2 results?

**We thank the reviewer for drawing our attention to this fact. The text was inserted into the manuscript (lines 365-366).**

Again, what is the conclusion?

**The conclusion was inserted into the text (lines 375-377).**

-   Discussion:

o    Line 404: which antioxidant? GPX1 only? (maybe SOD2?).

**We thank the reviewer and confirm that the antioxidant enzymes were duly inserted (line 410).**

o   Line 423: The levels of triglyceride in E T-Lep (close to E HV) and Y T-Lep seems quite different to me. Could the author test this? Which comparison and which test was performed in Table S1?

**We thank the reviewer for his careful analysis. There was no significant difference between the groups E T-Lep versus Y T-Lep (*P* = 0.5588). As we analyzed E HV *versus* Y T-lep, the result was not significant either (*P* = 0.3272). Data were analyzed by the Kruskal-Wallis method followed by Dunn’s post-testing multiple comparisons. Upon review of the table, we identified a typing mistake, that was duly corrected.**

o    Paragraph starting Line 442: This link between MTB and *M. leprae* is too implicit and does not bring insights to the point of the paper. Please develop.

**The reviewer is right. We hope the sentence inserted into the text is appropriate (lines 461-462).**

o    Paragraph starting Line 454: Again, the statements in this paragraph are too implicit. Please develop.

**We thank the reviewer for the remark and confirm that the paragraph was duly developed in the manuscript (lines 464-471).**

o    Line 463: The authors mention the lower synthesis ability of antioxidant enzymes in E L-Lep but I also see similar levels in E T-Lep. Could the authors comment on this observation?

**We thank the reviewer for raising this concern. If we compare E L-Lep to Y L-Lep, the expression of antioxidant enzymes in skin lesions are at similar levels, regardless of the leprosy clinical form. Nevertheless, our purpose was to compare, except as refers to GSR, the differences were significant.**

o    the last paragraph is quite confusing (especially line 477 and 478). How to draw a conclusion of L-Lep patients from an observation in T-Lep patients?

**The reviewer is right. A typing mistake induced us to error, and the sentence was corrected (lines 494 and 495).**

**--------------------**
<b>Summary and General Comments</b></br></br>

Use this section to provide overall comments, discuss strengths/weaknesses of the study, novelty, significance, general execution, and scholarship. You may also include additional comments for the author, including concerns about dual publication, research ethics, or publication ethics.

If requesting major revision, please articulate the new experiments that are needed.

Reviewer #1: The manuscript describes an investigation of the role of oxidative stress on leprosy pathogenesis in elderly individuals as compared to young affected and a sample of non-affected. Results suggest that the increased oxidative damage related to aging may alter leprosy pathogenesis; the authors suggest that antioxidant therapy may benefit elderly leprosy patients.

The well written manuscript reports interesting and relevant research much needed in leprosy, still a major public health burden in several countries. Several of the authors have an extensive history of contribution to the advance on the understanding of the molecular basis of leprosy pathogenesis, most of whom based at a highly reputed research center with optimal infrastructure for high quality basic research. The results are interesting, add considerably to the body of knowledge in leprosy and are of potential interest of the readers of PLOS NTD.

Upon careful reading of the manuscript, the following few major and minor concerns were raised, to which comments by the authors will be greatly appreciated:

**We thank the reviewer for the nice assessment of our paper.**

Major concerns:

The authors claim to have performed the Ridley & Jopling classification, and the results are used to stratify leprosy patients into groups for comparison; however, this is very difficult today given that several of the parameters originally described by R&J are no longer available, such as the Mitsuda reaction, for example. Can the authors comment on the criteria used to define patients as displaying LL, BL, BT and TT leprosy?

**We thank the reviewer and agree with his comments. The clinical presentation of lesions is paramount, and our staff includes specialized dermatologist to identify them. Moreover, the diagnostic is confirmed by histopathological analysis of lesions, performed by experienced pathologists and bacteriological index of slit skin smears (as recommended by WHO), and evaluates the disability grade and the lepromin test (Mitsuda reaction). To define R&J, we use clinical, histopathological and bacteriological parameters. Last, we keep a demographical and clinical database containing data from all the patients and contacts, such as education level, etc.**

There is no description of the sub-sample of non-affected individuals, described by the authors as "healthy volunteers". A healthy volunteer may be considerably different than a leprosy-free individual, and this may be particularly important if the variables involved (oxidative stress) may be impacted by several types of diseases and conditions. Can the authors kindly expand on how has this sub-sample been characterized?

**In our experimental design, we assessed two different groups of non-leprosy individuals selected. These groups were characterized as control groups, and, except as refers to histopathological analysis and cutaneous immunofluorescence, all the other analyses were performed with both groups. The individuals presented similar social and demographic features, and, most importantly, the fact of living in a leprosy endemic area. In addition, they shared the same characteristics as to gender, age and social class as the studied patients. As to the oxidative damage, the reviewer may observe, in the figure 1E, that the protein carbonyl levels were lower in the groups of healthy volunteers, regardless of age.**

The finding of different levels of SOD2 expression in skin samples of young vs. elder lepromatous patients is interesting considering that variants of this gene, located at the same genomic locus as PARK2 – a well-known leprosy susceptibility gene - have been previously associated with leprosy (Ramos et al., J. Inf. Dis, 2016); please consider using that information in the discussion.

**We thank the reviewer for drawing our attention to the fact. We discussed the issue in the manuscript, and the reference was duly inserted (lines 415-419).**

Minor comments:

Sometimes the reference to young and elder gets confusing as if refers to leprosy affected patients or non-affected. Please consider revise and if necessary, clarify throughout the text;

**We thank the reviewer for the comment, and, to avoid any misunderstanding, whenever we shall refer to patients or to healthy volunteers, we shall let it quite clear in the manuscript.**

The authors often include *p*-values in the text, which is quite uninformative if not associated with the actual values of the parameters under comparison; please consider including to increase the information content;

**We thank the reviewer for his comment and clarify that any reference to *P* value relates to statistical analysis performed, regardless of its significance.**

Line 294, the sentence would be clearer if stating that "... (Fig1B-1D) for L-Lep leprosy patients and for the HV group."

**We thank the reviewer for the comment and believe that the alteration in the description order of figure 1 contributed to a clearer understanding (line 296).**

Some parts of "results" seem to be better fitted into discussion (for example – and not limited to - lines 305-311 and 327-329), please consider reorganizing;

**We thank the reviewer. The text was insert into discussion section (lines 401-403).**

There are a few minor typos along the manuscript, easy to fix upon a careful revision.

**We thank for the careful analysis of the manuscript and inform that the requested corrections were duly performed.**

**Remark: the main table was inserted at the end of the manuscript (line 831), and the supplementary tables were uploaded as separate files, as requested by the Editor. The funding sources were duly rectified in the “funding” section.**
